# Do not discount the diagnosis of VKH based on race: self-reported race and ethnicity of patients with Vogt-Koyanagi-Harada disease in a predominantly white population

**DOI:** 10.1186/s12348-023-00329-2

**Published:** 2023-03-29

**Authors:** Gábor Gy. Deák, Anjum F. Koreishi, Debra A. Goldstein

**Affiliations:** 1 Department of Ophthalmology, Feinberg School of Medicine, Northwestern University, Chicago, IL USA; 2 Department of Ophthalmology and Optometry, Medical University of Vienna, Vienna, Austria

**Keywords:** VKH, Vogt-Koyanagi-Harada, Uveitis, Demographics, United States

## Abstract

**Background:**

We examined the racial and ethnic distribution of patients with Vogt-Koyanagi-Harada disease (VKH) in a Midwestern US population through a retrospective chart review of patients with VKH seen in a tertiary referral centre between 2012 and 2017. All patients were diagnosed by one uveitis specialist (DAG). We identified 32 patients with VKH seen during this time period. The mean age at diagnosis was 37.7 ± 15.7 years, 7 were male, 25 female. Mean follow-up was 36.7 ± 21.7 months. Nine patients reported themselves as White non-Hispanic, (28.1%), 9 as Black/African-American (28.1%), 2 as Asian (6.3%) and 9 as Hispanic or Latino (28.1%). Three patients (9.4%) were of Middle-Eastern origin. The 2010 census results for race and ethnicity in the state of Illinois were: 71.5% White, 14.5% Black/African-American, 4.6% Asian, and 6.7% as Some Other Race. From the total population 15.8% reported themselves as Hispanic or Latino (of any race).

**Conclusions:**

VKH was much more frequent among white non-Hispanic patients (28.1%) and Black/African-American patients (28.1%) in our patient population than in previous reports from the US (3–14% and 4–23% respectively). While Hispanic patients in this series were over represented in the VKH population compared with the overall census data, the percentage of VKH patients in this series who were White non-Hispanic and Hispanic was the same. The diagnosis of VKH should be considered in any patient with the appropriate clinical features, regardless of race or ethnicity.

## Background

Vogt-Koyanagi-Harada (VKH) disease is a rare ocular inflammatory condition. It was described by several different authors in the early 20^th^ century [1–3], and was later considered to be one single disease entity. The hallmark features of the acute phase of VKH are bilateral granulomatous panuveitis, serous retinal detachments and disk oedema. This phase may be followed several weeks or months later by a convalescent and a chronic phase typically characterised by chronic anterior uveitis, choroidal depigmentation (sunset glow fundus), diffuse choroiditis, vitiligo and poliosis. VKH is a clinical diagnosis as there are no specific diagnostic laboratory or imaging tests. The First International Workshop on Vogt-Koyangi-Harada disease suggested a set of diagnostic criteria, including the typical ocular findings seen in acute and late phases of the disease, as well as neurological signs such as meningismus, tinnitus or cerebrospinal fluid pleocytosis, and late dermatologic signs such as alopecia, poliosis and vitiligo [4].

Although VKH has been described in populations from different geographic locations, races and ethnicities, and no racial of ethnic factor is included in the current diagnostic criteria, VKH is still generally considered a disease of people with Hispanic, Asian or Middle-eastern origin. This can lead to delayed diagnosis and therapy in patients of other ethnicities [5]. We noted that many of our patients with VKH were not of the “typical” ancestry, and in some cases the referring physician had discounted the diagnosis, despite typical clinical features, because of the patient’s ethnicity. Thus, the aim of our study was to describe the racial and ethnic background of our patients with VKH seen at a single uveitis centre in the Midwestern United States.

## Methods

The study was a retrospective chart review of patients with Vogt-Koyanagi-Harada disease seen by the uveitis service of the Department of Ophthalmology, Northwestern University, Chicago, Illinois, USA between 2012 and 2017. The study protocol adhered to the tenets of the Helsinki agreement, and was approved by the institutional ethics committee. Eligible patients were identified through billing codes by the institution’s Electronic Data Warehouse, and were further confirmed by the investigators. All patients were diagnosed by one experienced uveitis specialist (DAG) using the revised diagnostic criteria for Vogt-Koyanagi-Harada disease [4]. Patients with complete, incomplete and probable VKH were included in the study. Patients in whom VKH was initially coded as a diagnosis, but in whom another diagnosis was ultimately reached were excluded. Some of the patients were first diagnosed by the same author (DAG) at a different institution, and were seen in follow-up at our institution. Because of this, age at diagnosis and age at baseline examination are presented separately.

Self-reported race and ethnicity were recorded either at check-in or by an ophthalmologist using a uveitis questionnaire at the baseline visit. Patients were allowed to select multiple races and ethnicities, and even sub specify their answer.

Demographic data of the general population of both the city of Chicago and the state of Illinois were gathered from the 2010 US Census conducted by the United States Census Bureau (available: factfinder.census.gov).

## Results

Forty two patients were identified for whom VKH was coded as a possible diagnosis. After reviewing all records, 32 patients with the diagnosis of VKH disease confirmed by a uveitis specialist were included in the analyses. 7 of the patients were male (22%) and 25 were female (78%); the mean age at baseline was 45.7 years. Age at diagnosis, available in 27 cases (84%), was 37.7 years. One patient had complete VHK with neurologic signs at the time of onset, and late skin lesions, 8 patients had incomplete VKH with neurologic signs and/or tinnitus at the time of onset and 23 patients had probable VKH with eye involvement only, based on the revised diagnostic criteria of VKH [4].

In our cohort 15 patients lived in the city of Chicago, and another 13 elsewhere in Illinois. One patient lived in North Carolina, one in Pennsylvania, and one in Indiana.

The racial and ethnic distribution of VKH patients in our cohort as well as the comparative U.S. Census results from 2010 for the city of Chicago and the state of Illinois are presented in Table 1.

**Table 1:** Racial and ethnic distribution of our cohort and the U.S. Census data. ^1^ Results from the 2010 census by the U.S. Census Bureau. ^2^ Hispanic or Latino origin among all the races [6].

	**VKH Cohort Percent (No)**	**Census Chicago** **Percent (No)**^**1**^	**Census Illinois Percent (No)**^**1**^
Asian	6.3% (2)	5.5% (147,164)	4.6% (586,934)
Black/African-American	28.1% (9)	32.9% (887,608)	14.5% (1,866,414)
Hispanic or Latino	28.1% (9)	28.9% (778,862)^2^	15.8% (2,027,578)^2^
White	28.1% (9)	45% (1,212,835)	71.5% (9,177,877)
White Middle Eastern	9.4% (3)	-	-
American Indian and Alaska Native	-	0.5% (13,337)	-
Some Other Race	-	13.4% (360,493)	6.7% (861,412)

## Discussion

Vogt-Koyanagi-Harada disease is typically reported in patients of certain races and ethnicities; most commonly those of Asian, Hispanic or Middle-Eastern origins as well as First Nations and Métis and Inuit [7–12].

One possible explanation for this is the higher number of melanocytes in certain tissues (such as skin, choroid, internal ear) in these populations, as the most widely accepted hypothesis for the pathogenesis of VKH disease is a CD4 + T-lymphocyte associated direct cellular response against melanocytes and probably free melanin [13].

We had noted that we saw more Caucasian and African-American patients with VKH than typically reported in the literature, so undertook a formal assessment of the race and ethnicity of our patients with VKH.

Publications from the United States report a high number of Hispanic patients in their cohorts. Beniz et al. from Los Angeles, California described 48 patients diagnosed with VKH. ^13^ 75% of patients were Hispanic, 10% White, and 4% Black. Rubsamen and Gass reported that of 26 patients with VKH seen at Bascom Palmer Eye Institute in Miami, Florida, 54% were Hispanic, 23% Black, 14% White, and 9% Asian [14]. In these US reports the prevalence of White and Black/African-American patients among VKH patients is lower than in our cohort, in which the prevalence of White, Black/African-American and Hispanic patients were each 28.1%. In our series both White and Black/African-American patients were overrepresented, and Hispanic or Latino ethnicity was underrepresented compared to other series from the US. A possible explanation for this might be the general racial and ethnical distribution of these US regions. In the 2010 census results the racial distribution and Hispanic origin in Los Angeles was: 49.8% White, 9.6% Black/African-American, 11.3% Asian, and 23.8% Some Other Race. 48.5% of people reported Hispanic or Latino origin (among all races). In Miami-Dade County, Florida 73.8% were White, 18.9% Black/African-American, 1.5% Asian, and 65.0% reported Hispanic or Latino origin Compared to Chicago both places have a much greater Hispanic population [6]. Even though VKH is more common in Hispanic patients, due to the higher percentage of White and Black/African-American patients in the Midwest, both of these groups are overrepresented in our cohort.

In our cohort complete VKH was the least common diagnosed form of VKH based on the revised VKH diagnostic criteria [4]. This is in line with other reports, and can be explained due to the fact that in this form both symptoms typical of the acute phase of the disease and skin findings- that develop in later stages- have to be present [9, 15]. Although in the literature incomplete VKH seems to be the most common form reported, in our cohort it was only the second most common form following probable VKH. One explanation for this could be that, the clinical diagnosis of VKH has become more common with the advent of better imaging, allowing the diagnosis to be made in the absence of systemic findings.

There are a number of caveats of our study. Most importantly are its retrospective nature and the relatively low number of patients. However, since VKH is an orphan disease, our patient population is still among the larger ones reported in the literature. Another drawback is the possibility of selection bias through our institution’s tertiary referral nature, and due to insurance issues. Unfortunately these issues cannot be overcome unless a state or nationwide registry system is instituted to register all patients diagnosed with an orphan disease. It is also difficult to directly compare the regional demographics from the exact catchment area of our patient population, as we are limited to city, county, and state census data.

## Conclusions

In summary, more than 50% of the patients with VKH in this series self-reported as White non-Hispanic or Black/African-American. This is a much higher number compared to data reported from other parts of the United States and from other countries. Vogt-Koyanagi-Harada should be considered in the differential diagnosis of patients with appropriate clinical features regardless of their race or ethnicity, and the lack of “typical” race or ethnicity should not delay appropriate management.

## Data Availability

The datasets used and/or analysed during the current study are available from the corresponding author on reasonable request.
